# Molecular Functions of Hydrogen Sulfide in Cancer

**DOI:** 10.3390/pathophysiology28030028

**Published:** 2021-09-20

**Authors:** Rodney E. Shackelford, Islam Z. Mohammad, Andrew T. Meram, David Kim, Fawaz Alotaibi, Stavan Patel, Ghali E. Ghali, Christopher G. Kevil

**Affiliations:** 1 Department of Pathology & Translational Pathobiology, LSU Health Sciences Center Shreveport, Shreveport, LA 71130, USA; mohammad.islam@lsuhs.edu (I.Z.M.); chris.kevil@lsuhs.edu (C.G.K.); 2 Head & Neck Oncologic/Microvascular Reconstructive Surgery Department of Oral & Maxillofacial/Head & Neck Surgery, Louisiana State University Health Sciences Center, Shreveport, LA 71130, USA; andrew.meram@lsuhs.edu (A.T.M.); david.kim@lsuhs.edu (D.K.); falota@lsuhsc.edu (F.A.); stavan.patel@lsuhs.edu (S.P.); gghali@lsuhs.edu (G.E.G.)

**Keywords:** hydrogen sulfide, H_2_S, cancer, cystathionine β-synthase, cystathionine γ-lyase, 3-mercaptopyruvate sulfurtransferase

## Abstract

Hydrogen sulfide (H_2_S) is a gasotransmitter that exerts a multitude of functions in both physiologic and pathophysiologic processes. H_2_S-synthesizing enzymes are increased in a variety of human malignancies, including colon, prostate, breast, renal, urothelial, ovarian, oral squamous cell, and thyroid cancers. In cancer, H_2_S promotes tumor growth, cellular and mitochondrial bioenergetics, migration, invasion, angiogenesis, tumor blood flow, metastasis, epithelia–mesenchymal transition, DNA repair, protein sulfhydration, and chemotherapy resistance Additionally, in some malignancies, increased H_2_S-synthesizing enzyme expression correlates with a worse prognosis and a higher tumor stage. Here we review the role of H_2_S in cancer, with an emphasis on the molecular mechanisms by which H_2_S promotes cancer development, progression, dedifferentiation, and metastasis.

## 1. Introduction

Hydrogen sulfide (H_2_S) is a colorless, corrosive gas with a characteristic rotten egg smell, known for over 300 years as an environmental toxin [1]. High H_2_S concentrations cause damage in many organs, including the brain, kidneys, and lungs. Its toxic mechanisms are usually attributed to cytochrome C oxidase, Na^+^/K^+^ ATPase, carbonic anhydrase, monoamine oxidase, and possibly ATR kinase inhibition [1,2,3,4]. While H_2_S has been identified in mammalian tissues for decades, it was not until 1996 that endogenous H_2_S synthesis and function as a biological modulator in humans was identified [5]. Presently, along with nitric oxide and carbon monoxide, H_2_S is recognized as the third gasotransmitter and functions as a master regulator of many different physiologic and pathologic processes [6,7]. For example, H_2_S functions in the cardiovascular, renal, central nervous, and digestive systems [5,8,9,10]. Dysregulated H_2_S function is also seen in many diseases, including metabolic and Down’s syndromes, cardiovascular disease, neurodegeneration, and cancer [11,12,13,14,15,16].

## 2. H_2_S Chemistry, Synthesis, and Molecular Mechanisms of Action

### 2.1. H_2_S Chemistry

The biochemistry of H_2_S is ancient and was likely essential in the development of life along with its antecedent, pre-biotic chemistry [17]. H_2_S is a weak diprotic acid, with first and second pKa values of 6.76 and 19 at 37 °C. At the physiologic pH H_2_S is ~70–80% HS^−^, ~20% H_2_S, with low concentrations of S_2_^−^ [18]. H_2_S readily passes through biological membranes allowing for efficient paracrine signaling, while HS^−^ has a high nucleophilicity, conferring chemical reactivity [19,20]. Biological membranes are not significant barriers to H_2_S diffusion, possibly due to the rapid equilibrium between HS^−^ and H_2_S [19]. The minus two oxidation state of the sulfur in H_2_S is the lowest possible sulfur oxidation state, making H_2_S an obligatory reductant [7]. H_2_S-oxidant interactions have been examined in many studies, where H_2_S is reported to have antioxidant effects [21,22,23,24]. Interestingly, H_2_S and H_2_O_2_ react too slowly to exert significant biologic effects [25,26]. Moreover, cellular H_2_S concentrations are low at 10–30 nM, compared to other thiol-based antioxidants, such as glutathione at 1–10 mM. Thus, under biologic conditions it is unlikely that free H_2_S has a quantitatively significant antioxidant role [27,28]. Most likely, a significant portion of H_2_S-mediated antioxidant activity is due to its induction of other cellular antioxidant defense systems, such as increasing cystine uptake, and cellular glutathione (GSH) and cysteine concentrations [7,21]. Additionally, free H_2_S exists in a thermodynamic equilibrium with many other sulfur species, including persulfides, polysulfides, and numerous sulfide metabolites, many of which are antioxidants [7]. In most tissues the H_2_S half-life is short, lasting only a few minutes [7,29].

### 2.2. H_2_S Synthesis

H_2_S is chiefly synthesized by three systems: (1) cellular enzymes, (2) nonenzymatic synthesis, and (3) production by the microbiome. Enzymatic H_2_S synthesis is based on the reverse transsulfuration pathway, where dietary methionine is converted into homocysteine. Cystathionine β-synthase (CBS) then catalyzes the condensation of homocysteine with cysteine, yielding cystathionine and H_2_S [30]. Cystathionine γ-lyase (CSE) catalyzes the conversion of cystathionine into α-ketobutyrate, cysteine, and NH_3_ [30]. CBS and CSE are both pyridoxal 5′-phosphate-dependent enzymes and can catalyze other substrates, depending on the specific substrate concentrations [7,31]. For example, CSE also catalyzes the conversion of L-cysteine to thiocysteine, pyruvate, and NH_3_. Thiocysteine in turn can non-enzymatically break down into pyruvate and H_2_S. H_2_S is also enzymatically synthesized by cysteine aminotransferase which converts cysteine to mercaptopyruvate, which 3-mercaptopyruvate sulfurtransferase (3-MST) in turn converts into H_2_S and pyruvate [7,31].

CBS, CSE, and 3-MST show distinct tissue and subcellular distributions and exert different contributions to specific cell/tissue H_2_S pools. 3-MST is primarily mitochondrial and synthesizes roughly 90% of brain H_2_S [6,32]. CBS exists within many tissues but is predominantly seen in the central nervous system and liver, while CSE is predominantly located in the vasculature [30,31]. Intracellular H_2_S exists as free H_2_S, acid-labile sulfide, and bound sulfane sulfur [29]. Free cellular H_2_S represents less than 1% of the potentially available sulfide, indicating that the endogenous sulfide pool likely has significant buffering capacity [7].

CBS, CSE, and 3-MST also synthesize polysulfides. In the brain, 3-MST synthesizes H_2_S_3_, which is known to alter the activities of proteins, such as KEAP1, PTEN, and GAPDH [33,34]. CSE overexpressing human A549 carcinoma cells have high polysulfide levels, while CSE knockout cells show significantly lower levels, and recently CSE-generated polysulfides have been shown to play a role in ovarian cancer [35,36]. Additionally, enzymatic kinetic analyses have shown that polysulfides are likely products of CBS and CSE activities [34]. Last, the enzyme cysteinyl tRNA synthetase produces protein hydropersulfides, which are involved in the regulation of mitochondrial biogenesis and bioenergetics, and many other cellular functions [37].

Nonenzymatic H_2_S synthesis also accounts for a portion of H_2_S synthesis and occurs by an iron and vitamin B_6_-mediated catalysis of thiol and thiol-containing compounds, such as cysteine [38]. Last, the mammalian microbiome regulates the bioavailability and metabolism of systemic H_2_S. For example, compared to conventionally housed mice, germ-free mice exhibit significantly lower plasma and gastrointestinal free H_2_S, and 50–80% lower plasma, adipose, and lung tissue bound sulfane sulfur. Tissue CSE activity was also reduced in many of the organs in germ-free mice, while tissue cysteine levels were elevated [39].

### 2.3. Molecular Mechanisms of H_2_S Activity

Based on its chemistry, the molecular mechanisms of H_2_S reactivity have been placed into three categories: (1) chemical interfacing/scavenging with reactive oxygen and nitrogen species, (2) chemical modification of protein cysteines to persulfides, and (3) binding to and/or redox reactions with metal centers [7,20]. Additionally, H_2_S may chemically reduce protein disulfides. The biochemical significance of this mechanism is currently unknown, and if it occurs, it is likely highly specific to the chemical milieu of the protein disulfide [40].

## 3. H_2_S and Cancer

Recently, a complex and intricate role for H_2_S has been identified in cancer. Below we discuss the role of H_2_S in several common human malignancies and discuss the functions of H_2_S in the initiation and promotion of cancer.

### 3.1. H_2_S and Colorectal Cancer

Colon cancer is the second most lethal and third most common cancer worldwide [41]. The role of H_2_S in cancer was demonstrated in 2013 when increased cytoplasmic and mitochondrial CBS expression was detected in seven colorectal carcinoma resections, compared to benign adjacent colonic tissues. CSE and 3-MST levels were unchanged. Several malignant colon cancer-derived cell lines similarly showed high CBS expression. Tumor tissue lysates incubated with L-cysteine and L-homocysteine also produced 6-fold more H_2_S than lysates from benign tissue. CBS knockdown or pharmacologic inhibition by aminooxyacetic acid (AOAA) reduced cell proliferation and H_2_S synthesis in the HCT116 colon cancer cell line, but not in the slower-growing nonmalignant NCM356 cell line. CSE knockdown or pharmacologic inhibition by DL-propargyl glycine (PAG) affected neither HCT116 cell proliferation nor H_2_S synthesis. AOAA also suppressed the migration and invasion of HCT116 cells, while NaHS, an H_2_S donor, enhanced these events [42]. Low to moderate concentrations (0.1–1 mM) of the CBS allosteric activator S-adenosyl-L-methionine also increased HCT116 cell proliferation, bioenergetics, and H_2_S synthesis for up to twelve hours, an event not seen with CBS knockdown [42,43].

CBS knockdown or AOAA inhibition also attenuated mitochondrial function, suppressing basal respiration, ATP production, and the spare respiratory capacity. CBS knockdown similarly reduced glycolysis, in part through reduced glyceraldehyde 3-phosphate dehydrogenase (GAPDH) activity. Cystathionine failed to stimulate cell proliferation or cellular bioenergetics, indicating that H_2_S and not cystathionine affected these cell behaviors. CBS, but not CSE knockdown, also significantly reduced the growth and volume of HCT116 cell xenografts in Nu/nu Balb/C female mice, with a concomitant reduction in CD31-immunopositive blood vessel density, the prevalence of larger blood vessels, and blood vessel branching. Additionally, the direct injection of AOAA into the xenograft tumor parenchyma reduced peritumor blood flow, while administration of the CBS substrate L-cysteine increased this flow [42,43].

Further studies revealed that premalignant colonic tubular adenomas (low-grade epithelial dysplasia) exhibited increased CBS expression, indicating that increased CBS expression is an early event in colon cancer [44]. Lentiviral vector-mediated CBS expression in the nonmalignant NCM356 colon cell line increased H_2_S synthesis, reverse transsulfuration pathway flux, cell proliferation, migration, invasion, mitochondrial bioenergetics, and glycolysis. CBS expressing NCM356 cells also formed larger tumors in athymic nude mice compared to vector only bearing cells. Whole transcriptome RNA sequencing was employed to compare the CBS expressing NCM356 cells to those carrying the vector alone. CBS expression caused a metabolic shift towards anabolic metabolism, increasing the gene expression patterns related to glycolysis, hypoxic responses, extracellular matrix formation, cell adhesion molecule production, and epithelial to mesenchymal transition (EMT), resulting in a gene expression pattern that overlapped with an oncogenic phenotype [44].

These studies were extended when HCT116 cells were treated with 300 μM of the 3-MST inhibitor 2-[(4-hydroxy-6-methylpyrimidin-2-yl)sulfanyl]-1-(naphthalen-1-yl)ethan-1-one (HMPSNE). HMPSNE suppressed 3-MST, CBS, the H_2_S-degrading enzymes ethylmalonic encephalopathy 1 protein, and rhodanese expression, suggesting that 3-MST-derived (but not CBS/CSE-derived) H_2_S exerts transcriptional regulatory actions in these cells [45]. HMPSNE treatment also increased mRNA and protein expression of the epithelial markers E-cadherin and tight junction protein ZO-1. Concomitantly, mRNAs for the mesenchymal markers fibronectin, Wnt3, β-catenin, and cadherin-associated protein β1, the transcription factors Snail1, Twist1, Sp1, and Sp3, and β-catenin, and both the ATP citrate lyase (ACLY) protein and mRNA expression were suppressed. ACLY is a key enzyme in acetyl-CoA synthesis associated with Wnt signaling and is implicated in the colon cancer EMT [45]. ACLY enzymatic activity was not induced by H_2_S; however, H_2_S partially reversed its inactivation by reactive oxygen species (ROS). Moreover, the slow H_2_S donor GYY4137 activated the ACLY gene promotor through the induction of the Sp3 transcription factor. Lastly, HMPSNE treatment also attenuated cell migration, consistent with EMT inhibition [46]. Taken together, the above studies indicate that H_2_S promotes the progression of colon cancer from preneoplastic low-grade dysplastic lesions to metastatic, dedifferentiated colon cancer.

The inhibition of H_2_S synthesis increases the sensitivity colon cancer cells to chemotherapeutic agents, while chemotherapy resistance is associated with increased H_2_S synthesis. For example, the treatment of the colon cancer cell lines HT-29 and DLD-1 with AOAA increased their sensitivity to 5-fluorouracil (5-FU), lowering cell colony formation, Bcl-2 expression, and viability, while increasing cell S-phase accumulation, apoptosis, cytochrome C release, and Bax expression. In a murine tumor xenograft model, DLD-1 cells stably over-expressing luciferase were injected into mice, allowing the bioluminescent noninvasive imaging of the cells. AOAA and 5-FU treatments in this model both lowered tumor growth, while the two combined synergistically suppressed tumor growth. AOAA also suppressed the target of 5-FU, thymidylate synthase. Further analysis revealed that CBS-synthesized H_2_S suppressed the colon cancer tumor suppressor miR-215-5p, increasing both thymidylate synthase and epiregulin (an EGFR signaling activator) [47]. AOAA treatment also increased the sensitivity of the colon cancer cell line oxaliplatin, significantly reducing the IC_50_ value, and GSH, Bcl-2, total caspase-9, and total caspase-3 expression, while increasing oxaliplatin-induced apoptosis, ROS levels, cleaved caspase-9, cleaved PARP, Bax, and p53 expression. In murine xenograft studies, AOAA and oxaliplatin reduced colon cancer cell growth, while the combination significantly reduced tumor volume compared to either agent alone [48].

**Figure 1:**
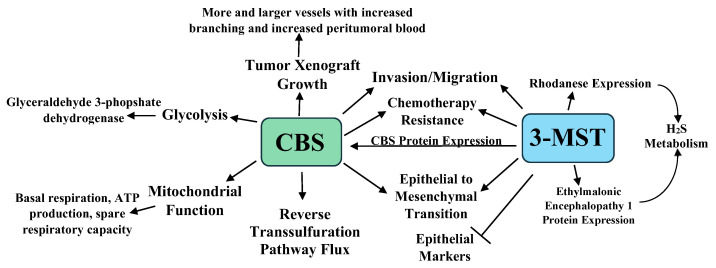
A summary of the roles of CBS, 3-MST, and H2S in colon cancer growth, progression, metastasis, and chemotherapy resistance [41,42,43,44,45,46,47,48,49].

In an interesting study, HCT116 cells were cultured for six months with increasing 5-FU concentrations in media maintained at 30 μM of 5-FU. When challenged with 3–100 μM of 5-FU, the parental cells exhibited lower viability and proliferation, while the 5-FU resistant cells showed no viability or proliferative changes. Western blot analyses revealed that CBS, 3-MST, and rhodanese increased by 49%, 63%, and 107% in the 5-FU resistant cells, with higher CBS and 3-MST enzymatic activities in these cells [49]. The 5-FU resistant cell line showed increased glutamate oxaloacetate transaminase 1 and cytochromes p450 CYP1A2 and CYP2A6 expression. The p450 inhibitor phenylpyrrole sensitized the 5-FU resistant cells to 5-FU treatment, while the sensitivity of the parental cell line 5-FU was not increased by this treatment. Lastly, the 5-FU resistant cells were resistant to AOAA treatment, showing less viability and proliferation suppression than the parental cell line when treated with AOAA and 5-FU. Taken together, the above data indicate that increased CBS and 3-MST protein levels and enzymatic activities play a role in chemotherapy resistance [49]. The roles of CBS, 3-MST, and H_2_S in colon cancer are summarized in Figure 1.

### 3.2. H_2_S and Ovarian Cancer

Ovarian cancer carries a 1.39% lifetime risk in women and due to its often-advanced stage at presentation, it is one of the most lethal gynecologic malignancies [50]. A tissue microarray analysis of 210 epithelial ovarian tumors revealed increased CBS expression compared to benign counterpart tissue. Moderate to strong CBS immunoreactivity was associated with a serous histology and a higher tumor grade, although significant expression was also seen in early-stage tumors. Additionally, of six ovarian tumor cell lines examined, four showed significant CBS protein and mRNA expression, while two showed high CSE expression compared to a benign epithelial ovarian cell line. CBS siRNA knockdown or AOAA treatment of the CBS-expressing ovarian cancer cells lines resulted in a significant suppression of cell proliferation (measured by ^3^H-thymine incorporation) and a lowered viability. CBS siRNA knockdown also resulted in a significant reduction in the total cellular GSH (GSH + GSSH) and increased RelA/p65 expression (a subunit of NF-κB), with concomitant increased ROS and p53 protein expression. Interestingly, culturing the cells in 5 mM Na_2_S resulted in a 20% increase in cell viability and culturing in 2 mM of GSH-containing media resulted in a complete recovery of cell viability, indicating a link between CBS-synthesized H_2_S, GSH levels, and cell viability. Lastly, siRNA CBS KD also increased the sensitivity of the ovarian cancer cell line to cisplatin, lowering the IC_50_ from 13.1 to 7.9 μM [51].

The A2780 ovarian cancer cell line showed both cytoplasmic and mitochondrial CBS expression. CBS knockdown in these cells significantly enhanced MitoSOX fluorescence, indicating increased superoxide. Additionally, citrate synthase activity was also decreased with increased AOAA concentrations, suggesting that CBS knockdown inhibited mitochondrial oxidative capacities. To confirm this, siRNA CBS knockdown and scrambled RNA (scRNA)-treated cell mitochondrial bioenergetics were analyzed [51]. CBS knockdown decreased both basal and maximally stimulated mitochondrial respiration. These changes were accompanied by an increase in the NAD/NADH and ADP/ATP ratios and ROS levels, and a decrease in total ATP [51]. Taken together, these data demonstrate a role of CBS in the maintenance and regulation of mitochondrial function in ovarian cancer [51].

To extend these observations, the mitochondrial fusion and fission apparatus proteins MFN1, MFN2, Drp1, OPA1, and Fis1 were examined in two ovarian cancer cell lines (CP20 and OV90) with scRNA or CBS siRNA knockdown [52]. Of the five proteins, only MFN2 expression was down-regulated by CBS knockdown. Interestingly, the Mitotracker Red CMXRos staining of these cells revealed that the scRNA cells had fused and elongated the mitochondrial morphology, while the siRNA CBS knockdown cells displayed a predominantly spherical mitochondrial morphology and more unbranched mitochondria with a shorter length. Additionally, CBS or MRN2 siRNA knockdown significantly lowered the mitochondrial oxygen consumption rate and the membrane potential, cell proliferation, ATP production, and spare respiratory capacity. The treatment of siRNA CBS knockdown cells with the slow H_2_S donor GYY4137 increased the mitochondrial oxygen consumption rate, suggesting that CBS-generated metabolites regulate mitochondrial function. No changes in the autophagy markers or PGC1α were detected in the CBS knockdown cells. Similarly, the mitochondrial genome number did not change with CBS knockdown. Based on this, it was concluded that CBS knockdown induced mitochondrial fragmentation, but not autophagy by MFN2 suppression. Additionally, CBS maintained mitochondrial health through promoting fusion, ATP synthesis, and MFN2 stabilization [52].

CBS silencing did not affect MRN1 or MRN2 mRNA expression. Immunoprecipitation analyses revealed that CBS silencing increased MFN2 ubiquitination, resulting in its destabilization and degradation. Support for this came from the observation that treatment with the proteasome inhibitor MG132 rescued MFN2 expression with CBS silencing. The JNK kinase is activated by oxidative stress, resulting in its phosphorylation of MFN2, followed by MFN2 ubiquitination. Treatment of CBS silenced cells with either the antioxidant mitoTEMPO or the JNK inhibitor SP600125 rescued MFN2 expression [52]. Thus, CBS maintains mitochondrial function by attenuating ROS levels, an event required to preserved MFN2 protein expression [52]. The clinical relevance of CBS and MFN2 expression in ovarian cancer was examined in the clinically annotated mRNA data from The Cancer Genome Atlas (Gene Expression Omnibus) databases. In the quantitative analysis for Kaplan–Meier overall survival curves, a high expression of CBS and MFN2 conferred a poor overall survival of patients, with CBS and MFN2 expression showing a significant association in the same patient cohort [52].

**Figure 2:**
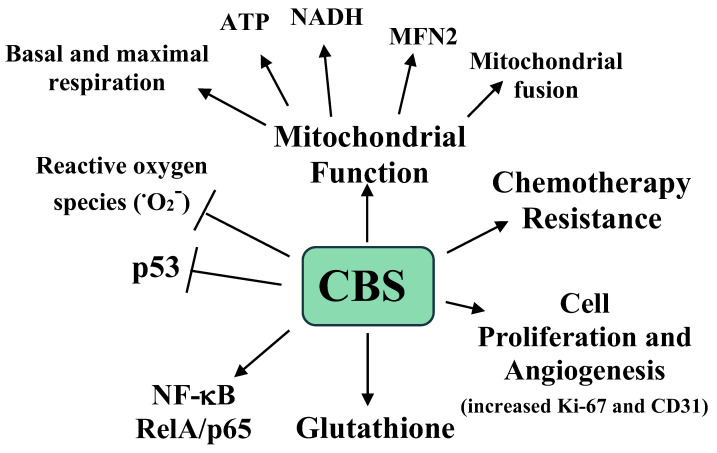
A summary of the roles of CBS and H2S in ovarian cancer growth, progression, metastasis, mitochondrial, function, and chemotherapy resistance. O_2_^−^ is the superoxide radical [51].

The effects of CBS expression on tumor growth were examined in a previously developed murine orthotopic model of ovarian cancer. Mice were interperitoneally injected with the ovarian A2780 tumor cell line and after one week the mice were injected with neutral liposomes containing either control siRNA or CBS siRNA twice weekly, with or without cisplatin [52]. After four weeks the mice were sacrificed, and the tumors were analyzed. The mice receiving the CBS siRNA had a 40% lower tumor weight, a 70% reduction in tumor nodules, and lower proliferation and blood vessel formation, as measured by Ki-67 and CD31 immunostaining, respectively [52]. Interestingly, with cisplatin, the siRNA treated mice showed a small and nonsignificant reduction in tumor weight, while the mice treated with CBS siRNA plus cisplatin exhibited a 90% reduction in tumor weight compared to the mice treated with siRNA only [51]. The roles of CBS and H_2_S in ovarian cancer are summarized in Figure 2.

### 3.3. Breast Cancer

Breast cancer is the most common cause of cancer death in women worldwide [53]. A tissue microarray was employed to analyze CBS expression in 60 breast cancer cases compared to adjacent benign tissue. CBS expression was significantly increased in breast cancer, and increased further with higher breast cancer stages, with the highest expression in metastatic disease. CBS mRNA and protein levels were also higher in breast cancer cell lines compared to benign mamillary epithelial cells, with the cancer cells lines producing 2 to 4-fold more H_2_S and 64-fold more cystathionine than the benign cells. CBS knockdown in the breast cancer cell lines lowered H_2_S and cystathionine synthesis, but not cell growth in culture [54].

CBS knockdown accompanied by co-culture with activated macrophages caused significant cell growth suppression. Additionally, following three weeks growth in murine xenograft models, CBS siRNA knockdown in breast cancer cells resulted in lower tumor volumes compared to cells without knockdown (8 mm^2^ vs. 125 mm^2^). Lentiviral-mediated increased CBS expression in breast cell lines that expressed undetectable CBS levels caused a 4.5-fold increase in H_2_S synthesis, easily detected CBS protein, and no growth suppression when co-cultured with activated macrophages (something seen without increased CBS). CBS knockdown and co-culture with activated macrophages increased the levels of the 4-hydroxylnonenal (4-HNE) and malondialdehyde (MDA) breast cancer cell lines. Co-incubation with the slow-releasing H_2_S donor GY4137 reduced 4-HNE and MDA levels by 40–50% and increased cell growth by 60–80%. Cell fractionation experiments revealed that CBS localized to the breast cancer cell membrane, demonstrating that CBS conferred resistance to macrophage-generated ROS by plasma membrane H_2_S synthesis [55]. Interestingly, cystathionine is also highly enriched in breast cancer due to high CBS expression. Cystathionine has been shown to protect breast cancer cells from ROS-induced damage, chemotherapy-induced apoptosis, and promote mitochondrial and endoplasmic reticulum homeostasis in breast cancer cells. Thus, CBS may also promote breast cancer via the production of this cancer-protective oncometabolite [54].

Similarly, CSE expression was analyzed in 15 breast cancer cases and compared to benign adjacent tissues. CSE mRNA and protein levels were increased in breast cancer compared to benign tissue and increased in breast cancer cell lines compared to a benign mamillary epithelial cell line. siRNA CSE knockdown in MCF7 breast cancer cells lowered H_2_S synthesis, cell proliferation, EdU incorporation, and cell migration, while increasing apoptosis and S phase cell arrest. CSE overexpression increased H_2_S synthesis, EdU incorporation, and cell migration. Stat3 knockdown inhibited cell proliferation and migration, while increased Stat3 expression enhanced these capacities. An analysis of the CSE promoter demonstrated that phospho-Stat3 binds to the CSE promoter and increases CSE protein expression [56].

**Figure 3:**
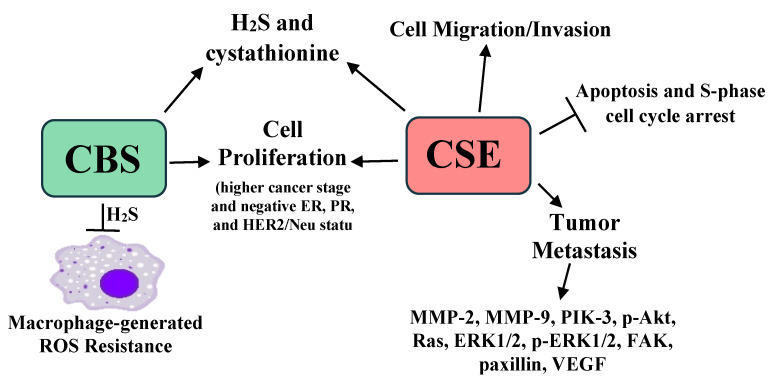
A summary of the roles of CBS, CSE, and H2S in breast cancer growth, progression, metastasis, chemotherapy resistance, and resistance to macrophage-generated ROS [54,55,56,57,58,59].

High breast cancer CSE promotes breast cancer metastasis. A separate analysis of 30 breast cancer cases revealed that CSE was: (1) higher in breast cancer than adjacent benign breast tissue, (2) increased in stage III cancers compared to stage II, (3) highest in metastatic disease, and (4) higher in ER, PR, and HER2/Neu immunonegative tumors (triple negative/basaloid breast cancer). Interestingly, triple negative breast cancer has a high recurrence and metastasis risk [57]. In the MBA-MB-231 breast cancer cell line, siRNA CSE knockdown reduced cell migration, invasion, and proliferation, while CSE overexpression enhanced these events. When MBA-MB-231 cells were injected into the tail veins of nude mice and lungs subsequently histologically examined four weeks later, a 75% pulmonary metastasis rate was seen. However, the metastatic rate was 12.5% with CSE knockdown. CSE knockdown in these cells also lowered the expression of angiogenesis and metastasis-associated/promoting proteins, including MMP-2, MMP-9, PIK-3, Akt, p-Akt, Ras, Raf, ERK1/2, phospho-ERK1/2, FAK, paxillin, and the vascular endothelial growth factor, while increased CSE expression increased the expression of these proteins [58]. Not surprisingly, a recently developed CSE inhibitor, I194496, lowered animal model metastases, while lowering the expression of the proteins listed above [59]. Taken together, these data indicate that CBS and CSE are increased in breast cancer and play a significant role in breast cancer development, progression, and metastasis. The roles of CBS, CSE, and H_2_S in breast cancer are summarized in Figure 3.

### 3.4. Bladder Cancer

Bladder cancer is the 10th most common cancer worldwide, with the highest rates found in Europe, Australia, and North America, and it causes approximately 115,000 deaths each year [60]. In 94 patients who had had undergone a transurethral resection or a radical cystectomy for urothelial bladder cancer (UBC) all showed moderate to strong immunoreactivity for CBS, CSE, and 3-MST in the UBC, compared to little or no staining in a benign urothelium. The expression levels and the production of H_2_S in UBC tumor lysates increased with an increase in the tumor grade and stage [61]. Exogenous H_2_S also significantly increased cell proliferation and invasion, and MMP-2 and MMP-9 protein expression in the EJ human bladder cancer cell line. These events were increased with higher exogenous H_2_S dosages, suggesting that H_2_S increases the proliferative, invasive, and metastatic capacities of UBC [62].

### 3.5. Renal Cancer

Clear cell renal carcinoma (ccRCC) accounts for ~2.4% of malignancies and is characterized by a Von Hippel–Lindau protein (VHL)-deficiency, resulting in pseudohypoxic, glycolytic tumors [63]. A comparison of two VHL-deficient ccRCC cell lines (769-P and 786-O cells) to a VHL-wild-type ccRCC cell line and benign HK-2 renal cells revealed significantly higher H_2_S synthesis in the 769-P and 786-O cells. Although the levels of CBS, CSE, and 3-MST were the same in the different cells, enzyme inhibition preferentially suppressed the proliferation, metabolism, and survival of VHL-deficient ccRCC cell lines. Last, the systemic inhibition of H_2_S synthesis in a chorioallantoic membrane angiogenesis model significantly decreased vascularization in the VHL-deficient ccRCC cell xenografts. Taken together, these data support the idea that VHL loss results in pseudohypoxia and low mitochondrial oxidation, hence a low mitochondrial H_2_S catabolism, and subsequent increased cellular H_2_S [64].

In a tissue microarray study of 21 benign renal cortex and 94 ccRCC samples, CBS showed increasing expression in ccRCC Fuhrman grades II–IV. Thus, CBS expression may play a role in ccRCC progression [65]. Interestingly, in a study of 21 ccRCC tissue samples compared to benign renal tissues from the same individuals, 66% of the pair samples showed stable CBS expression, with the remaining samples exhibiting suppressed tumor tissue CBS levels. CSE expression was suppressed in all but three of the tumors compared to benign tissue, and 3-MST was suppressed in 70% of the tumors and was unchanged in 30% of the tumors compared to benign tissue. The authors concluded that the H_2_S-synthesizing enzymes showed varying expression in ccRCC [66]. The reason for this difference may lie in the fact that first study employing microarray technology did not compare benign/malignant tissues from the same patients [66].

### 3.6. Prostate Cancer

Prostate cancer is the most common male cancer in the US and is the 6th leading cause of male death worldwide [67]. In an elegant study, human PC3 prostate cancer cells were injected into the ventral prostate of male nude mice and allowed to grow for 40 days. Tumor cells were then isolated from the prostate and bone marrow, with the later representing metastatic disease. Seven sets of prostate/bone marrow metastatic PC3-derived cells were analyzed by microarray. CSE, but not CBS or 3-MST, was up-regulated in five of the metastatic cells vs. the cell pairs localized to the prostate. Two of these cell sets were further analyzed and the metastatic cells showed increased migration and invasive capacities, but a similar proliferation and similar GSH levels. The overexpression of CSE in the PC3 cells increased cellular H_2_S levels, migration, and invasion, while siRNA knockdown suppressed these events. Interestingly, the treatment of the cells with the irreversible CSE inhibitor PAG substantially reduced invasion, but not cell migration, raising the possibility that CSE enzymatic activity is only required for cell invasion. To explore this possibility, a CSE mutant with low enzymatic activity was generated (CSE^Q240E^) which had a decreased kinetic activity of ~70-fold. Cells carrying this mutation still exhibited cell migration but minimal invasion, indicating that CSE promotes cell migration by an enzymatic activity-independent mechanism [68].

To analyze human prostatic cancer CSE expression, 105 prostate tumors vs. 11 benign prostatic tissue samples were analyzed by microarray technology. CSE expression was significantly greater in stage III/IV prostate cancer than in stages I/II or adjacent non-tumor tissue. Patient survival and CSE expression was then correlated by using the TCGA PC RNA-seq dataset containing 469 prostate cancers and 50 benign tissue samples. CSE expression was higher in prostate cancer and at the eight-year follow-up, a significant correlation between high CSE levels and poor patient survival was identified. A similar association between CSE expression and poor survival was also identified in pancreatic adenocarcinoma and lower grade gliomas, implying a role for CSE in the progression of other cancer types [68].

Incongruously, in another study comparing prostate tumors and benign prostatic tissue, CBS expression was not changed, 3-MST was not detected, and CSE expression was lower in prostatic cancer. CSE expression was also lower in the prostatic tissue of aged mice compared to younger mice and was accompanied by a concomitant increase in proliferating cell nuclear antigen (PCNA) and cyclin D1 levels. Older CSE knockout mice also showed even higher PCNA levels. CSE expression was also lowered in prostatic cancer cell lines following antiandrogen-resistance development. Last, H_2_S treatment inhibited the binding of the androgen receptor to the prostate-specific antigen (PSA) and the androgen-responsive element due to the post-translational androgen receptor S-sulfhydration inhibiting the binding of the androgen receptor [69].

### 3.7. Thyroid

Thyroid cancer constitutes approximately 1% of human malignancies, making it the most common of the neuroendocrine malignancies. Approximately 85% of thyroid cancers are the well-differentiated papillary (PC) and follicular carcinoma (FC) subtypes. The few remaining common subtypes are anaplastic thyroid (AC) and medullary thyroid carcinomas (MC) [70]. A tissue microarray analysis revealed that CBS expression is increased in PC, FC, MC, and ACs, but not in benign thyroid follicular adenomas [71]. In another study CSE, but not CBS or 3-MST expression, was increased in thyroid PC. PC CSE levels were significantly increased with an increased tumor size, extrathyroidal extension, and with lymph node metastases, but not with patient gender, age, or tumor stage. Interestingly, sonic hedgehog signaling pathway molecules, sonic hedgehog, patched, and smoothened were also increased on PC, with significant increases seen in the same parameters as for CSE, suggesting interactions between CSE and the sonic hedgehog pathways [72]. The reasons for the differences in CBS and CSE expression in thyroid cancer shown in these studies likely lie in the different patient populations analyzed and the different experimental techniques used.

In a comparison of a benign human thyroid cell line to thyroid cancer cell lines, 25–50 μM NaHS increased CBS, sulfide:quinone oxidoreductase, p-PI3K, p-AKT, p-mTOR, H-RAS, p-RAF, p-MEK1/2, and p-ERK1/2, and rhodanese protein expression, while enhancing cell proliferation, migration, and viability in the cancer, but not benign cell lines. All of these events were inhibited by 200 μM NaHS in the cancer cell lines [73]. Furthermore, 1.4–2.8 mg/kg/day NaHS promoted tumor growth and blood vessel formation in human thyroid carcinoma xenograft tumors models, while 11.2 mg/kg/day NaHS inhibited these events. In summary, this study demonstrated that: (1) the ROS/PI3K/Akt/mTOR and RAS/RAF/MEK/ERK signaling pathways function in conjunction with H_2_S to promote thyroid cancer cell growth and (2) H_2_S exerts cancer cell growth-promoting or -inhibiting effects depending on the dosage [73].

### 3.8. Pulmonary Adenocarcinoma

An analysis of 20 pulmonary adenocarcinomas and benign matched lung tissue samples showed increased H_2_S levels and increased CBS, CSE, and 3-MST in the malignant tissue. Similar results were also found in three lung cancer adenocarcinoma cell lines (A549, NCI-H522, and NCI-H1944 cells) compared to nonmalignant lung epithelial cells (BEAS 2B cells). An analysis of mitochondrial DNA (mtDNA) stability in the lung cancer and adjacent benign lung tissue revealed that the tumor mtDNA had significantly fewer DNA breaks and a higher mtDNA copy number. The same analysis of the lung cancer and BEAS 2B cells revealed higher DNA damage in the lung cancer cells. A MitoSOX analysis of the A549, NCI-H522, and BEAS 2B cells revealed higher ROS in the tumor cells. A549 cell treatment with AOAA or siRNA knockdown of CBS, CSE, or 3-MST in the presence of mild chronic oxidative stress increased mtDNA breaks, an event blocked by the mitochondrial-specific H_2_S donor AP39. Incubation with serine or homocysteine for 24 h resulted in no changes in mtDNA breaks, ruling out their accumulation as a mechanism of changes in mtDNA breaks [74].

To further these studies, the mitochondrial-specific DNA repair enzyme EXOG which forms complexes with APE1, PolG, and Lig3 was analyzed by proximity ligation assay with and without 300 μM of AOAA treatment in A549 cells. AOAA significantly reduced the complex formation, while simultaneous treatment with AP39 restored the complex formation. Mass spectrometric analysis of EXOG revealed that EXOG Cys 76 was post-translationally sulfhydrated by oxidized H_2_S metabolites. Co-immunoprecipitation assays with wild-type and mutant EXOG C76A with APE1 showed that Cys 76 was required for the EXOG/APE1 complex formation, and the complex formation was enhanced by cell NaHS treatment. This NaHS-induced enhancement was not seen with the mutant EXOG C76A. These data indicate that H_2_S plays a central role in lung cancer mitochondrial DNA repair [74]. Similar to other studies, the inhibition of H_2_S synthesis in the A549 cells, but not the BEAS 2B cells, reduced all major mitochondrial bioenergetic parameters. This reduction was rescued by AP39 treatment in the A549 cells. Lastly, in a murine xenograft model, A549 cells showed reduced growth with AOAA or irinotecan treatment, and significantly reduced tumor growth with both treatments, demonstrating that H_2_S synthesis inhibition potentiated the tumor-suppressing effects of this chemotherapy agent [74].

### 3.9. Melanoma

Melanoma is the deadliest skin cancer and the 19th most common cancer worldwide [75]. In a study of six compound nevi, four junctional nevi, and four dysplastic nevi, all were positive for CSE, variably positive for 3-MST, and negative for CBS. An analysis of one normal human epidermal melanocytic (NHEM) cell line and four melanoma cell lines showed a very low expression of all three enzymes in the NHEM cell line, with the melanoma cell lines showing mildly increased CSE, and no changes in CBS and 3-MST expression. CSE overexpression in the melanoma cell line with the lowest CSE expression (A375 cells), inhibited cell proliferation by 30%. CBS and 3-MST overexpression had no effect [76].

Garlic (*Allium sativum*) has a long history of medicinal use and contains several H_2_S-donating compounds, such as diallyl trisulfide (DATS) [77,78]. DATS, similar to the H_2_S donor GYY4137, releases H_2_S relatively slowly, while H_2_S donors such as Na_2_S and NaHS instantaneously disassociate in solution, releasing H_2_S very rapidly, unlike the slower, steady-state H_2_S production by the three H_2_S-synthesizing enzymes [79]. Since an increased CSE expression inhibited cell proliferation, the melanoma cell line was treated with DATS and GYY4137 to examine the effects of slow H_2_S release on melanoma cell line viability [76]. Treatment of the A375 cell line with these H_2_S donors inhibited proliferation, induced apoptosis, and caused a G_0_/G_1_ phase arrest [76].

Melanoma growth is partially dependent on constitutive NF-κB activation and promotor binding [80]. Interestingly, DATS and GYY4137 treatment inhibited NF-κB DNA binding and anti-apoptotic gene expression via the inhibition of IκBα degradation. Similarly, treatment with these H_2_S donors reduced the expression of the melanoma cell lines p-AKT and p-ERK. Lastly, in a murine xenograft melanoma animal model, subcutaneously injected melanoma cells exhibited a 51% growth reduction with the oral administration of the CSE substrate L-cysteine. This effect was negated by the oral co-administration of the CSE inhibitor PAG. Taken together, these data indicate that CSE exerts inhibitory effects on melanoma growth through the inhibition of NF-κB and the growth-promoting signal transduction pathway [76].

### 3.10. Oral Squamous Cell Carcinoma

Head and neck cancers are the 6th most common malignancies worldwide, and 70% are squamous cells carcinomas (SCC) [81]. The comparison of 15 oral SCCs to adjacent benign tissue revealed a roughly 3-fold increase in CBS, CSE, and 3-MST in the SCC. Increased Stat3, p-Stat3, MitoNEET, nicotinamide phosphoribosyl transferase, hTERT, and MAPK were also identified. It is interesting that increased p-Stat3 was identified in SCC, as this transcription factor increases CSE expression in breast cancer [56]. SCC and adjacent benign H_2_S concentrations were measured directly by an immediate punch biopsy followed by quick-freezing in liquid N_2_ and H_2_S analysis by the monobromobimane method coupled with reverse phase-HPLC [29]. H_2_S concentrations were significantly (~13%) increased in the SCC compared to the adjacent benign tissue [16]. Although H_2_S-synthezing enzymatic activities were not measured, the 3-fold increased expression of all three enzymes suggests that tumor H_2_S synthesis was much higher than 13%. Thus, tumor synthesized H_2_S is likely being transferred to other portions of the sulfur pool, contributing to oncogenesis [36].

## 4. Protein Sulfhydration and Cancer

Protein sulfhydration is a post-translational protein modification where a sulfur atom is added to a reactive protein cysteine, forming an -SSH or a persulfide group. The cysteine persulfide formation involves an oxidized cysteine derivative reacting with a sulfide or sulfide oxidation products. Protein sulfhydration exerts predominately inhibitory effects, with most activating events due to the persulfidation-induced inhibition of a negative regulator [7]. Here we will review several well characterized cancer-related sulfhydrated proteins.

### 4.1. NF-κB

NF-κB is a family of dimeric transcription factors that are activated by a large number of stimuli and play an important role in immune responses, inflammation, and cancer [82]. In the prostate cancer PC3 cell line, NaSH treatment increased cell invasion and NF-κB p65 cysteine 39 sulfhydration, as measured by a modified biotin switch assay. Maximal p65 cysteine 39 sulfhydration was seen at NaSH concentrations of 10 nM to 10 μM, indicating that the sulfhydration occurred at physiologically relevant donor concentrations [82]. Conversely, CSE knockdown decreased this sulfhydration and cell invasion. PC3 cells with p65^C38S^ mutant expression exhibited attenuated NaSH-induced invasive capacities and abolished sulfhydration. The re-expression of wild-type p65 expression rescued these events. In a murine xenograft animal model, the p65^C38S^ mutant showed significantly fewer metastases than wild-type p65. Taken together, these data indicate that the sulfhydration of the p65 subunit Cys plays a central role in promoting prostate cancer metastasis [68].

### 4.2. GAPDH

The sulfhydration of GAPDH cysteine 150 was measured by a biotin switch assay. While wild-type CSE elicited GAPDH sulfhydration, the catalytically inactive mutant CSE did not. Similarly, GAPDH sulfhydration was identified in the livers of CSE wild-type, but not CSE knockout mice. The treatment of HEK293 with NaHS over a 3 to 100 μM concentration increased GAPDH enzymatic activity by up to 700% [83]. As this is a high concentration of a fast H_2_S releasing donor, the physiological significance of this event requires further study, ideally with a lower concentration of a slow-releasing H_2_S donor. A role for GAPDH in cancer comes from the observation that CBS knockdown in colon cancer cell lines lowers GAPDH enzymatic activity [42].

### 4.3. Lactate Dehydrogenase (LDHA)

LDHA converts pyruvate to lactate and is increased in colon cancer, where it contributes to tumor proliferation and bioenergetics. Treatment of HCT116 cells with GYY4137 increases mitochondrial function and glycolysis in these cells. GYY4137 treatment increases LDHA cysteine 163 sulfhydration, increasing its activity. GYY4137 treatment did not increase LDHA activity with the LDHA^C163A^ mutation. GYY4137 treatment also increased the total cellular lactate with wild-type LDHA expression, but not with LDHA^C163A^ expression. Last, LDHA^C163A^ expression increased cellular sensitivity to ROS. Based on these results, it was concluded that the sulfhydration of LDHA cysteine 163 plays a role in H_2_S-promoting colon cancer [84].

The number of sulfhydrated proteins in cancer is rapidly growing and there are far more than the ones listed above. Undoubtedly, this area of research will grow and many more sulfhydrated proteins that are important in cancer will be identified.

## 5. Polysulfides and Cancer

High CBS and CSE expression confer a poor prognosis in ovarian cancer [36,52]. Surface-enhanced Raman spectroscopy of 186 stage III or IV ovarian cancers indicated that high CSE expression correlated with a poor prognosis, tumor cisplatin resistance, and increased tumor polysulfides. Similarly, high CSE expression in ovarian tumor cell lines increased cisplatin resistance due to increased polysulfide synthesis. CSE knockdown increased the sensitivity of ovarian tumor cells to cisplatin and was accompanied by a concomitant increase in histone H2AX phosphorylation and decreased polysulfides. In in vitro experiments hydrogen polysulfides reduced cisplatin-induced DNA damage, with less damage seen with increasing sulfur atom numbers in each polysulfide [36]. Interestingly, the tumor suppressor gene product PTEN is inactivated by polysulfides where sulfane sulfur is added to an active site cysteine, ablating PTEN phosphatase activity [85]. It is likely that this and many other polysulfide-related events play an under-appreciated role in cancer.

## 6. H_2_S-Associated Tumor Markers in Bodily Gas and Fluids

Multiple studies have shown increased H_2_S and related sulfur compounds in cancer patients compared to control subjects. For example, increased H_2_S occurs in the headspace vapor of gastric contents in patients with gastro-esophageal cancer, while increased H_2_S and methanethiol were identified in the flatulence and exhaled air of colon and lung cancer patients [86,87,88]. In men with prostate cancer, urine thiosulfate concentrations were 50-fold higher than men without cancer, suggesting that urine thiosulfate may have diagnostic value for prostatic cancer in men with low PSA and negative digital rectal exams. Men with benign prostatic hypertrophy had only a 5-fold increase in urine thiosulfate, differentiating hypertrophy from cancer [89]. Cysteine, homocysteine, and cystathione were also elevated in the urine of men with recurrent prostate cancer, while urine cystathionine and sarcosine concentrations correlated with prostate cancer stage [90,91,92]. High plasma homocysteine levels have been identified in individuals with several malignancies, including endometrial, esophageal, SCC, prostate, colorectal, and breast cancers [93]. Lastly, endogenous H_2_S has been used as a cancer biomarker and as a way to detect cancer cells in mice [94,95]. These studies indicate that H_2_S and related sulfur compounds can be elevated in bodily gas and fluids and may have utility in cancer diagnostics. Specifically, H_2_S and related sulfur compounds could be used in cancer detection though the measurement of compounds such as plasma and urine thiols, or employed to monitor the effectiveness of cancer treatment, remission induction, and the early detection of recurrence.

## 7. H_2_S and Nuclear DNA Repair

Recently, a role for H_2_S in DNA repair has been identified, where ATR kinase inhibition resulted in lower cellular H_2_S levels. Low cellular H_2_S concentrations increased ATR kinase activity measured by CHK1 phosphorylation, while high H_2_S concentrations suppressed ATR ser-435 phosphorylation, a marker of ATR kinase activation [4,96]. Increased ATR-CHK1 pathway activity has been identified in several tumor types [97,98,99,100]. Additionally, increased ATR protein, phospho-ATR, and phospho-CHK1 expression confers a poor prognosis in breast, bladder, and ovarian cancers [98,99,100]. As the ATR kinase regulates H_2_S concentrations, it is possible that increases in ATR and CHK1 result in increased H_2_S synthesis. Additionally, targeted ATR inhibition is now being explored in cancer therapy [101]. Possibly, this cancer therapy approach may work, in part, through the suppression of H_2_S synthesis [95]. These possible cancer-related events should be explored.

## 8. The Immunomodulatory Role of H_2_S in Cancer

H_2_S exerts complex and powerful immune system modulating effects in both normal and pathologic conditions, often showing impaired functions at very low and high H_2_S concentrations [102]. Presently, there is some evidence that H_2_S-induced immune modulation plays a role in cancer [55]. As previously discussed, in breast cancer CBS localizes to the cancer cell membrane, where CBS-derived H_2_S protects the cancer cells from activated macrophage-generated ROS [55]. Additionally, in an interesting study melanoma cell-bearing mice were injected with a vehicle or the vehicle with DATS and analyzed for melanoma growth, splenic myeloid-derived suppressor cells (MDSCs), dendritic cells, and T cells [103]. The MDSCs function by suppressing tumor-specific T lymphocytes, contributing to malignant progression. DATS injection inhibited melanoma growth and lowered the frequency of splenic, blood, and tumor micro-environment MDSCs, while increasing CD8 T cells and dendritic cells. DATS treatment also significantly reduced the immune-suppressive activity of the MDSCs, restoring T cell function and T cell-mediated tumor growth suppression, demonstrating that H_2_S donation modulates tumor growth through immune system regulation [103]. These studies indicate that H_2_S can both enhance and suppress tumor growth through immune system modulation.

## 9. Conclusions, Issues, and Future Directions

**Figure 4:**
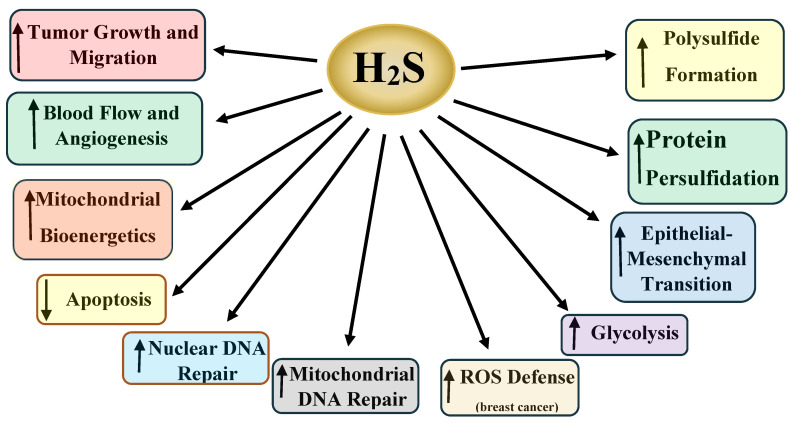
A synopsis of some of the cancer-promoting effects of H2S in cancer. Together, these events function together to increase tumor grade and stage, increase metastatic potential, and confer chemotherapy resistance.

The summary presented here is incomplete due to the enormous body of research on H_2_S and cancer, where H_2_S promotes a plethora of cancer-related effects (vide supra, summarized in Table 1 and Figure 1, Figure 2, Figure 3 and Figure 4). Several areas in this research should be addressed, while other promising results require further examination.

First, many of the pharmacologic tools used in H_2_S research have flaws. For example, while AOAA is commonly used as a CBS inhibitor, it also inhibits CSE, 3-MST and over thirty other cellular enzymes, greatly complicating the experimental results [104]. Similar sub-optimal specificity is seen with many other H_2_S synthesizing enzyme inhibitors, such as β-cyano-alanine [105]. The commonly used pharmacologic H_2_S donors, Na_2_S and NaHS, also very rapidly disassociate in solution, likely resulting in high transient, non-physiologic H_2_S concentrations which do not reflect normal or even pathologic conditions [105]. Thus, advancing H_2_S research necessitates the development of more specific H_2_S-synthesizing enzyme inhibitors and the use of slow-release H_2_S donors that better reflect the slow and steady H_2_S enzymatic synthesis [96,105].

Secondly, an under researched area is the functional differences between the H_2_S synthesizing enzymes. For example, mutant CSE in prostate cancer cells resulted in low cellular invasive capacities, but did not affect cell migration, showing that the CSE-generated H_2_S was only one aspect of CSE-initiated activities [68]. Careful analysis of the functions of the H_2_S-syntheszing enzymes, including the mutation of different portions of the enzymes, should elucidate exactly what role each portion of the enzymes has in regulating cellular functions and in promoting cancer.

Third, polysulfides are a new and exciting area of cellular biology [18]. Presently there are several papers showing a role for polysulfides in cancer [36,85]. Undoubtedly, further research will show that polysulfides play an important and underappreciated role in cancer. Fourth, epigenetics plays a major role in cancer [106]. Presently, there are no data on H_2_S cancer epigenetics. It is very likely that in cancer, H_2_S will alter the epigenome in the promotion of cancer.

Lastly, while not covered in this review, targeted therapies directed towards H_2_S metabolism in cancer are a promising area in cancer treatment [96,107]. H_2_S synthesis inhibition and H_2_S donators have been shown to induce cancer cell death, while often sparing benign cells. Both of these processes are currently being explored, with and without concomitant chemotherapy [73,96,107]. A better understanding of the role of H_2_S in cancer will undoubtedly facilitate the development of better cancer therapies.

## Figures and Tables

**Table 1:** A summary of the expression patterns of CBS, CSE, and 3-MST in several different human malignancies and synopses of the findings.

Tumor Type	H2S Synthesizing Enzyme Expression	Comments	References
Colon Cancer	CBS increased	Higher colon cancer CBS promotes cancer growth, mitochondrial bioenergetic activity, and increased glycolysis, migration, invasion, and chemotherapy resistance. While not increased, 3-MST expression promotes colon cancer EMT.	[42,43,48,49]
Ovarian Cancer	CBS and CSE increased	Increased CBS and CSE promote events including cancer growth, more active mitochondrial bioenergetics and morphologic integrity, migration, invasion, chemotherapy resistance, and a poor prognosis. Data also indicates a role for polysulfides in ovarian cancer.	[36,51,52]
Breast Cancer	CBS and CSE increased	Increased CBS and CSE promote cell growth, migration, and chemotherapy resistance. Membranous CBS protects cells from macrophage-derived ROS and confers a worse prognosis. CSE promotes breast cancer metastasis.	[39,56,58]
Bladder Cancer	CBS, CSE, and 3-MST increased	H2S likely promotes bladder cancer cell proliferation and invasion, and MMP-2 and MMP-9 protein expression. H2S synthesis in tumor lysates positively correlates with tumor stage and grade.	[61,62]
Renal Cancer	Most studies show suppressed or unchanged expression	H2S appears to promote tumor growth. The present studies show contradictory results, possibly based on analysis methods employed.	[64,65,66]
Prostate Cancer	Increased or decreased CSE in different studies	CSE promotes cell proliferation, migration, invasion, and poor patient survival. CSE promotes cell migration by an enzymatic activity-independent mechanism. Another study shows that CSE suppression promotes prostatic cancer.	[68,69]
Thyroid Cancer	CBS and CSE increased	Different studies show increased CBS or CSE. NaHS promotes thyroid cancer proliferation, migration, and cell viability.	[71,72,73]
Pulmonary adenocarcinoma	CBS, CSE, and 3-MST increased	H2S promotes cell proliferation, mitochondrial bioenergetics, and mitochondrial DNA repair.	[74]
Melanoma	Mildly increased CSE expression	CSE expression inhibited melanoma cell growth and H2S donors increased apoptosis by NF-κB inhibition.	[76]
Oral squamous cell carcinoma	CBS, CSE, and 3-MST increased	Direct measurements of tumor H2S concentrations revealed 13% higher H2S than in adjacent benign oral squamous epithelium.	[16]

## Data Availability

Not applicable.
